# Protocol to evaluate mouse brain spatial cell type-resolved transcriptomic discoveries using 10× Visium spatial transcriptomics and FLEX scRNA-seq

**DOI:** 10.1016/j.xpro.2025.104277

**Published:** 2025-12-27

**Authors:** Mara S. Burns, Ricardo Miramontes, John C. Reidling, Ryan G. Lim, Leslie M. Thompson

**Affiliations:** 1 Department of Neurobiology & Behavior, University of California, Irvine, Irvine, CA 92617, USA; 2 Institute for Memory Impairments and Neurological Disorders, University of California, Irvine, Irvine, CA 92617, USA; 3 Department of Biological Chemistry, University of California, Irvine, Irvine, CA 92617, USA; 4 Department of Psychiatry & Human Behavior, University of California, Irvine, Irvine, CA 92617, USA

**Keywords:** cell biology, single cell, sequencing, RNA-seq, molecular biology, gene expression, neuroscience

## Abstract

Understanding changes in gene expression and cell-cell signaling among spatial regions in diseased tissues adds critical biological information to understanding mechanisms. Here, we present a protocol to investigate molecular transcriptional drivers within intact murine tissue using 10× Genomics Visium spatial transcriptomics and 10× Genomics FLEX single-cell RNA sequencing (scRNA-seq) data. We describe steps for collecting mouse brain tissue from multiple ages, processing samples, and mounting the tissue. We then detail procedures for staining, FLEX tissue section collection, fixation, dissociation, and cell storage.

For complete details on the use and execution of this protocol, please refer to Burns et al.^1^

## Highlights

• Mouse brain collection at multiple time points for spatial transcriptomics and scRNA-seq
• Steps for fresh frozen spatial tissue processing with Visium and FLEX scRNA-seq
• Processing of postnatal day 0 mouse brain tissue for sequencing

## Abstract (editor highlights)

Publisher’s note: Undertaking any experimental protocol requires adherence to local institutional guidelines for laboratory safety and ethics.

## Graphical abstract

**Figure:**
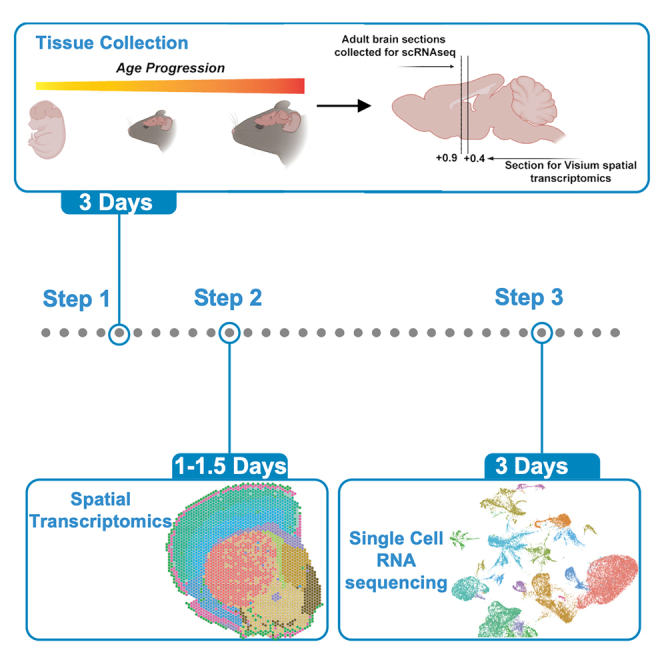

## Before you begin

Spatial transcriptomics allows mapping of regional gene expression patterns and altered signaling that occurs in pathological states compared to unaffected controls.^1^^,^^2^^,^^3^^,^^4^^,^^5^^,^^6^ Integration of single cell/nuclei sequencing (scRNAseq) with spatial data then allows further understanding of cell type specific regional changes.

In this protocol, we describe the specific steps to use 10× Genomics Visium (version 1) and FLEX scRNAseq (singleplexing and multiplexing) on fresh-frozen mouse brain tissue from non-transgenic and a Huntington’s disease (HD) mouse model, R6/2, at postnatal day 0 (P0), 4-week and 12-weeks (adult) of age. Our primary regions of focus were the striatum and cortex; however, the protocol can be altered according to mouse brain regions of interest. This methodology allows accurate integration of single cell sequencing with spatial sequencing information from matched samples of comparable regions in control and disease modeled mice. Further optimization might be required for variations in tissue type, mouse genotype related anomalies, brain regions or mouse age. For Visium, we recommend practicing mounting sectioned tissue into an 8 × 8 mm square to ensure confidence in tissue placement on the final slide. Timing on the graphical abstract is flexible based on personal preference and technical skill. We also estimated timing based on all the steps that proceed the major steps described in this protocol including probe hybridization for FLEX scRNAseq, library construction and sequencing for both sequencing methods.

Before beginning spatial sequencing, review 10× Genomics Visium Spatial Gene Expression for Fresh Frozen protocols related to appropriate fresh-frozen tissue preparation through tissue freezing and embedding, cryosectioning onto a Visium Gene Expression Slide, staining with immunofluorescence antibodies and imaging with an appropriate microscope. It is important to note that tissue optimization of permeabilization conditions for a tissue of interest is required since total mRNA maps where the gene activity is occurring and mRNA released during permeabilization binds to slide capture probes. Next steps are library construction generated from the cDNA and sequencing. Downstream analysis involve software analysis with Space Ranger and Loupe Browser.

Furthermore, before beginning FLEX scRNAseq, review Tissue Fixation & Dissociation for Chromium Fixed RNA Profiling and depending on if multiplexing or singleplexing samples, follow protocol for probe hybridization and sequencing.

### Innovation

This protocol advances spatial transcriptomics methods by integrating longitudinal state-of-the-art whole transcriptome data at spatial and single cell levels using multiple time points. We have optimized many of these steps from the 10× Genomics protocols to ensure high RNA integrity and efficient staining of Visium tissue sections before proceeding with library construction and sequencing, as well as dissociation of fragile tissue sections using liberase for fixed adult versus postnatal brain tissue to maximize nuclei/cell yield and quality. Furthermore, the use of matched mouse brains for these sequencing methods enables direct integration of spatial and cell-type-resolved transcriptomes, enhancing spatial resolution and biological interpretability of regional transcriptional changes to answer questions related to disease with a brain region focus on striatum and cortex.

### Institutional permissions

All experimental procedures were in accordance with the Guide for the Care and Use of Laboratory Animals of the NIH and animal protocols were approved by Institutional Animal Care and Use Committees at the University of California Irvine (UCI), an AAALAC accredited institution. In this study, we used R6/2 mice (Jax strain 006494 B6CBA-Tg(HDexon1)62Gpb/3J carrying CAG 120 ± 5) and control non-transgenic littermates at postnatal day 0, 4-weeks and 12-weeks of age.

**Figure 1:**
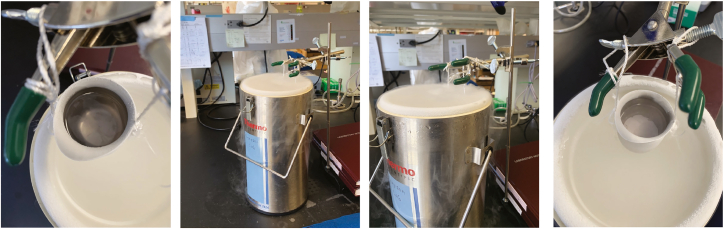
Setup of liquid nitrogen workstation for freezing mouse brain tissue Add isopentane to the metal beaker and place over liquid nitrogen container until a thin layer of ice forms on the bottom of the beaker.

### Preparation for tissue embedding with isopentane freezing method

**Timing: 30 min before perfusion and collection of tissue**

1. Set up a workstation and gather supplies for tissue embedding, including Tissue Tek Optimal Cutting Temperature (O.C.T.) compound, labeled cryomolds that are appropriate size for brain tissue, dry ice or liquid nitrogen, isopentane, a container suitable for making an isopentane bath, and forceps.
  a. To set up a liquid nitrogen workstation, assemble a Bel-Art Economy Support Stand, Three-Prong Extension and metal beaker (Figure 1).
2. At least 10 min prior to freezing the tissue in O.C.T., prechill isopentane over liquid nitrogen dewar, or dry ice if no access to liquid nitrogen.
  a. Attach metal beaker to Three-Prong Extension.
  b. Fill metal beaker about two-thirds full of isopentane, sufficient to submerge the tissue, and hover over liquid nitrogen dewar about an inch.

***Note:*** There should be a thin frozen layer on the bottom of the metal beaker (Figure 1). If left for too long, the isopentane will fully freeze, thus, remove metal beaker from dewar to allow to thaw so only the frozen layer is at the bottom.

3. Have a pre-chilled box in the −80°C freezer for keeping the samples once they are frozen for long term storage.
4. Prepare 4% Paraformaldehyde (PFA) fixative for the right hemisphere for immunohistochemistry protein validation.

## Key resources table

**Table:** 

REAGENT or RESOURCE	SOURCE	IDENTIFIER
**Antibodies**		

Chicken anti-GFAP (1:200 dilution)	Abcam	Cat# ab4674, RRID:AB_304558
Mouse anti-NeuN (1:200 dilution)	Millipore	Cat# MAB377, RRID:AB_2298772
Donkey anti-Chicken IgY (H+L) Highly Cross Adsorbed Secondary Antibody, Alexa Fluor 488 (1:200 dilution)	Thermo Fisher Scientific	Cat# A78948, RRID:AB_2921070
Donkey anti-Mouse IgG (H+L) Highly Cross-Adsorbed Secondary Antibody, Alexa Fluor 555 (1:200 dilution)	Thermo Fisher Scientific	Cat# A-31570, RRID:AB_2536180
DAPI Nuclear Counterstain	Fisher Scientific	Cat# 62248

**Chemicals, peptides, and recombinant proteins**		

Tissue Tek O.C.T. Compound	Fisher Scientific	Cat# 14-373-65
Liberase TL	Sigma-Aldrich	Cat# 05401020001
Liberase TH	Sigma-Aldrich	Cat# 5401135001
Isopentane (2-methylbutane)	Fisher Scientific	Cat# O3551-4
Paraformaldehyde	Fisher Scientific	Cat# 50980487
Sucrose	Sigma-Aldrich	Cat# 84097
Sodium Azide	Fisher Scientific	Cat# BP922I-500
Triton X-100 Solution, ∼10% in H_2_O	Sigma-Aldrich	Cat# 93443-100ML
SSC Buffer 20× Concentration	MilliporeSigma	Cat# S6639
MACS BSA Stock Solution	Miltenyi Biotec	Cat# 130-091-376
TruStain FcX PLUS (anti-mouse CD16/32) Antibody	BioLegend	Cat# 156603
AOPI staining solution	Nexcelom	Cat# CS2-0106
Nuclease-free water	Thermo Fisher Scientific	Cat# AM9937
Sodium dodecyl sulfate (SDS) solution	MilliporeSigma	Cat# 71736
Low TE Buffer	Thermo Fisher Scientific	Cat# 12090-015
Hydrochloric Acid Solution, 0.1N	Fisher Chemical	Cat# SA54-1
Protector RNase Inhibitor	MilliporeSigma	Cat# 3335399001
UltraPure Bovine Serum Albumin (BSA, 50 mg/mL)	Thermo Fisher Scientific	Cat# AM2616
Formaldehyde (37% by Weight/Molecular Biology), Fisher BioReagents	Thermo Fisher Scientific	Cat# BP531-25
Phosphate-Buffered Saline (PBS), 1× without Calcium and Magnesium	Corning	Cat# 21-040-CV
Dulbecco’s phosphate-buffered saline (DPBS), 1×, no calcium, no magnesium	Life Technologies	Cat# 14190-144
Tris Buffer, 1 M sterile solution, pH 8.0	VWR	Cat# E199-100ML
Gibco RPMI 1640	Thermo Fisher Scientific	Cat# 11875093
Glycerol for molecular biology, ≥99.0%	MilliporeSigma	Cat# G5516

**Critical commercial assays**		

Visium Spatial Gene Expression version 1	10× Genomics	PN-1000187 (4 reactions), PN-1000184 (16 reactions), RRID:SCR_023571
Visium Accessory Kit	10× Genomics	PN-1000194
Visium Spatial Tissue Optimization Slide & Reagent Kit	10× Genomics	PN-1000193
Visium Spatial Gene Expression Slide & Reagent Kit	10× Genomics	PN-1000187
Dual Index Kit TT Set A 96 rxns	10× Genomics	PN-100215
Chromium Fixed RNA Kit, Mouse Transcriptome	10× Genomics	PN-1000495 (4 reactions, single-plex), PN-1000496 (16 reactions, multi-plex)
Chromium Next GEM Chip Q Single Cell Kit (48 reactions)	10× Genomics	PN-1000418
Chromium Next GEM Single Cell Fixed RNA Sample Preparation Kit	10× Genomics	PN-1000414
RNeasy Mini Kit	QIAGEN	Cat# 74106

**Deposited data**		

Mouse spatial transcriptomics raw and analyzed data	Burns et al.^1^	NCBI GEO Number: GSE285858
Mouse snRNA-seq raw and analyzed data	Burns et al.^1^	NCBI GEO Number: GSE284468

**Experimental models: Organisms/strains**		

Mouse: B6CBA-Tg(HDexon1)62Gpb/3J; males, non-transgenic and R6/2 transgenic mice; P0, 4 weeks, 12 weeks	The Jackson Laboratory	RRID: IMSR_JAX:006494

**Software and algorithms**		

R (version 4.1.2)	R Core Team	RRID: SCR_001905
Space Ranger (version 2.0.0)	10× Genomics	RRID:SCR_025848
Loupe Browser 6 (Version 6.3.0)	10× Genomics	RRID:SCR_018555
Seurat (version 4.1.0)	Satija Lab	RRID: SCR_016341
Ingenuity Pathway Analysis (IPA)	QIAGEN	RRID: SCR_008653
Enrichr	Ma’ayan Laboratory	RRID: SCR_001575
CellRanger (version 7.1.0)	10× Genomics	RRID:SCR_017344
Harmony	Korsunsky et al.^7^	RRID:SCR_022206
CytoSPACE (version 1.1.0)	Vahid et al.^8^	N/A
STRING	STRING	RRID:SCR_005223
High dimensional weighted gene co-expression network analysis (hdWGCNA) (version 0.3.03)	Morabito et al.^9^	N/A
CellChat (version 2.1.2)	Jin et al.^10^ and Jin et al.^11^	RRID:SCR_021946
Allen Institute for Brain Science: Allen Brain Explorer	Wang et al.,^12^ Shi et al.,^13^ and Yao et al.^14^	atlas.brain-map.org
DevCCF	Kronman et al.^15^	https://kimlab.io/home/projects/DevCCF/index.html

**Other**		

Kimtech Science Kimwipes Delicate Task Wipers	Thermo Fisher Scientific	Cat# 06-666
C1000 Touch Thermal Cycler with 96-Deep Well Reaction Module	Bio-Rad	Cat# 1851197
SP Bel-Art Economy Support Stand, Bel-Art Products	VWR	Cat# 12000-070
VWR Talon Three-Prong Extension Clamps	VWR	Cat# 21570-302
Vannas Spring Scissors - 4 mm Cutting Edge	Fine Science Tools	Cat# 15018-10
Self-Standing Polypropylene Centrifuge Tubes, 50 mL, sterile	Corning	Cat# 430921
gentleMACS C Tubes	Miltenyi Biotec	Cat# 130-093-237
gentleMACS Octo Dissociator with Heaters	Miltenyi Biotec	Cat# 130-096-427
Pre-Separation Filters (30 μm)	Sysmex	Cat# 04-004-2326
Stainless Steel Metal Beaker	Thomas Scientific	Cat# 22A00H860
Agilent 2100 Bioanalyzer	Agilent	Cat# G2939BA
Razor Blades	Thermo Fisher Scientific	Cat# 12640
Standard Cryomold	VWR	Cat# 25608-916
Intermediate Cryomold	VWR	Cat# 25608-924
Biopsy Cryomold	VWR	Cat# 25608-922
EVOS Imaging System	–	
Keyence BZ-X810 Widefield Microscope	Keyence	Cat# 50061
Countess II FL Automated Cell Counter	Thermo Fisher Scientific	Cat# AMAQAF1000
Countess II FL Automated Cell Counting Chamber Slides	Thermo Fisher Scientific	Cat# C10228

## Materials and equipment

**Table:** 30% Sucrose (pH 7.0)

Reagent	Final concentration	Amount
Sucrose	30%	150 g
Sodium Azide	0.02%	0.1g
1× DPBS		Approx. 350 mL
**Total**		**500 mL**

• Use a clean, sterile beaker. Place magnetic stirrer inside.
• Add 150g sucrose. Add approximately 300 mL of 1× DPBS slowly to sucrose and turn on magnetic stirrer to start mixing. Mix until all sucrose goes into solution and solution is completely clear.
• Pour into graduated cylinder. Add 1× DPBS until you have a total of 500 mL DPBS. Pour into clean, sterile 500 mL bottle. Add 0.1g Sodium Azide to solution and mix thoroughly by inverting.
• Store at 4°C for up to 3 months.

**Table:** 2× Blocking Buffer for 4 reactions

Reagent	Stock concentration	Final concentration	Amount: 4× plus 20% more for pipetting error
Nuclease-free water			673.4 μL
SSC Buffer	20×	6×	878.4 μL
BSA	10%	4%	1174.2 μL
Triton X-100	10%	0.2%	58.6 μL
RNase inhibitor	40×	2×	146.4 μL
**Total**			**2928 μL**

• Keep all enzymes, buffers and Master Mixes on ice during setup and use.

**Table:** 1× Blocking Buffer for 4 reactions

Reagent	Amount: 4× plus 20% more for pipetting error
2× Blocking Buffer	161 μL
Nuclease-free water	119.6 μL
Moused TruStain FcX	9.2 μL
Ribonucleoside Vanadyl Complex	32.2 μL
**Total**	**322** μL

• Keep all enzymes, buffers and Master Mixes on ice during setup and use.

**Table:** Wash Buffer for 4 reactions

Reagent	Amount: 4× plus 20% more for pipetting error
2× Blocking Buffer	2415 μL
Nuclease-free water	1932 μL
Ribonucleoside Vanadyl Complex	483 μL
**Total**	**4830 μL**

• Keep all enzymes, buffers and Master Mixes on ice during setup and use.

**Table:** Primary antibody solution for 4 reactions

Reagent	1× Reaction	Amount: 4× plus 15% more for pipetting error
2× Blocking Buffer	25 μL	110 μL
Gfap (chicken)	1:200 of total	1.1
NeuN (mouse)	1:200 of total	1.1
RNase inhibitor	6.75 μL	29.7 μL
Nuclease-free water	Up to 50 μL	78.1
**Total**	**50 μL**	**220 μL**

• Keep all enzymes, buffers and Master Mixes on ice during setup and use.

**Table:** Secondary antibody solution for 4 reactions

Reagent	1× reaction	Amount: 4× plus 15% more for pipetting error
2× Blocking Buffer	25 μL	110 μL
488 donkey anti-chicken	1:200 of total	1.1 μL
555 donkey anti-mouse	1:200 of total	1.1 μL
DAPI	0.17 μL	0.75 μL
RNase inhibitor	6.75 μL	29.7 μL
Nuclease-free water	Up to 50 μL	77.35 μL
**Total**	**50 μL**	**220 μL**

• Keep all enzymes, buffers and Master Mixes on ice during setup and use.

**Table:** Mounting Medium

Reagent	Stock concentration	Final concentration	Amount: 200 μl (1 slide)
Glycerol	100%	85	170 μL
RNase Inhibitor	40×	2×	10 μL
Nuclease-free water			20 μL
**Total**			**200 μL**

• Make fresh and keep at 20°C.

**Table:** Fixation Buffer

Reagent	Stock concentration	Final concentration	Amount: Per 25 mg tissue (1 sample)
Nuclease-free water			791.9 μL
10× Concentration Fix & Perm Buffer	10×	1×	100 μL
Formaldehyde	37%	4%	108.1 μL
**Total**			**1000 μL**

• Keep all enzymes, buffers and Master Mixes on ice during setup and use.

**Table:** Tissue Resuspension Buffer

Reagent	Stock concentration	Final concentration	Amount: Per 25 mg tissue (1 sample)
PBS (Corning)			496 μL
Tris Buffer (pH 8.0)	1000×	50×	50 μL
BSA (50 mg/mL)	5%	0.02%	4 μL
RNase Inhibitor	40×	0.24×	6 μL
Nuclease-free water			444 μL
**Total**			**1000 μL**

• Keep all enzymes, buffers and Master Mixes on ice during setup and use.

**Table:** Quenching Buffer

Reagent	Stock concentration	Final concentration	Amount: Per 25 mg tissue (1 sample)
Nuclease-free water			875 μL
10× Conc. Quench Buffer	8×	1×	125 μL
**Total**			**1000 μL**

• Keep all enzymes, buffers and Master Mixes on ice during setup and use.

**Table:** Dissociation Solution

Reagent	Stock concentration	Final concentration	Amount: Per 25 mg tissue (1 sample)
RPMI			1920 μL
Liberase TH or Liberase TL	5 mg/mL	0.2 mg/mL	80 μL
**Total**			**2000 μL**

• Keep all enzymes, buffers and Master Mixes on ice during setup and then once ready, warm Dissociation Solution for 10 min at 37°C.

## Step-by-step method details

### Collection of mouse brain tissue

**Timing: 3 days if performing steps every day—1 day for collection of mice depending on breeding and ages, 1 day for tissue sectioning to perform RIN quality control analysis, 1 day for tissue sectioning to collect sections for scRNA-seq and mounting to Visium slide, 2–3 days to process fixed hemisphere**

These steps describe fresh frozen perfusion and mouse brain collection of postnatal day 0 (P0) and adult mice (4-weeks and 12-weeks of age), sample cryostat sectioning, determination of RIN value, collection of tissue sections for scRNAseq and placement of 10 μm tissue sections on the Visium slide. Refer to 10× Genomics Tissue Preparation Guide for additional details (protocol CG000240).

**CRITICAL:** Ensure that procedures involving live animals are approved by the IACUC and comply with governmental regulations.

***Note:*** Timing of steps depends on personal preference and technical ability. These are general guidelines and 3 days in total if performing every step back-to-back. There are not time constraints for certain steps.

1. Perfuse adult mice transcardially for brain collection. Timing is based on number of animals, but approximately 10 min per mouse.
  a. Inject adult mice with euthanizing overdose dosing pentobarbital. Ensure absence of tail or paw pinch reflex to confirm deep anesthesia.
  b. While under deep anesthesia, open thoracic cavity to expose the heart.
    i. Insert an 18-gauge perfusion needle through the left ventricle wall.
    ii. Connect the needle to a VWR pump that is connected to ice-cold sterile and RNAse free 1× DPBS.
    iii. Set the speed to approximately 5–7 mL/min.
    iv. Immediately puncture the right atrium with a needle tip or cut with scissors to perfuse animal and ensure euthanization.
  c. Once blood is cleared, which can be deduced by lightening in color of the liver, the needle should be removed, mouse decapitated, and the brain collected, which also lightens in color.
2. Collect postnatal day 0 (P0) mouse brains. Timing is based on number of animals, but approximately 5 min per P0 pup.
  a. Induce cryoanesthesia for P0 pups by placing on ice and decapitating using scissors.
  b. Dissect and remove whole brain by keeping the brain in a petri dish over ice. For fine and controlled dissection, use Vannas Spring Scissors (4 mm).
  c. Carefully blot the brain with a Kimwipe to remove excess blood and liquid.

**CRITICAL:** Step 2c is vital to ensure good RIN values and preserve RNA quality for P0 mouse brains.

**Figure 2:**
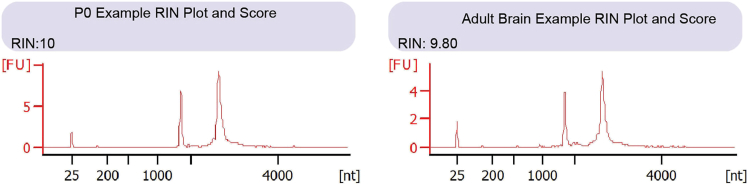
Example RIN electropherogram trace plot and score for P0 and adult brain samples A score of 10 indicates intact RNA while 1 is completely degraded. X axis depicts fragment size in nucleotides [nt] and y axis depicts fluorescence intensity in arbitrary units [FU].

**Figure 3:**
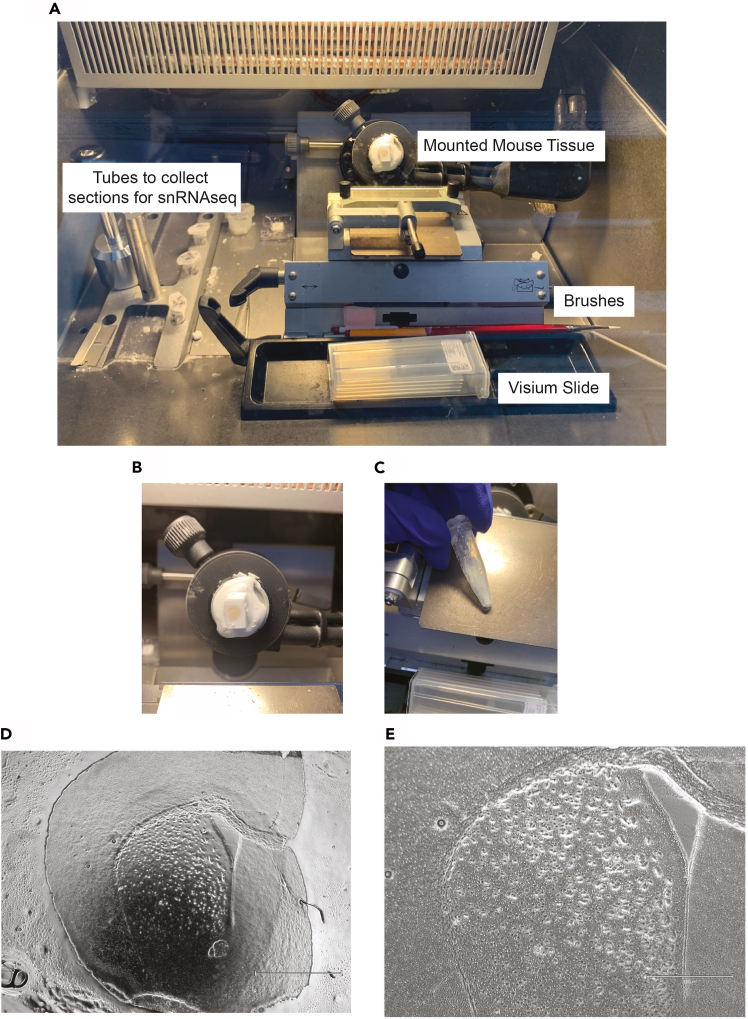
Mouse brain tissue collection cryostat setup (A) Cryostat workstation set up with mounted mouse brain tissue, brushes to smooth thin sections for mounting, Visium slide and tubes to collect sections for scRNAseq. (B) Score around mounted mouse brain to reduce O.C.T. around tissue. (C) Tube of collected brain tissue sections. (D) 2× EVOS image to visualize localization of brain region (scale bar = 1550 μm) and (E) 4× EVOS image (scale bar = 750 μm).

3. For any age of mouse, embed right (or left) hemisphere in Tissue-Tek O.C.T. by snap freezing over isopentane bath immersed in liquid nitrogen.
  a. Embed adult mouse brain in the Standard Cryomold or Intermediate Cryomold and embed P0 mouse brain in the Biopsy Cryomold.
  b. Use this hemisphere to proceed with 10× Visium.
  c. Long-term store at −80°C in a sealed container or plastic bag with desiccant to prevent condensation.
  d. Keep in −80°C at least 12 h then proceed to Step 5, however, samples can be stored for long term if desired.
4. Post-fixation of brain hemisphere for protein or RNASCOPE staining validation of findings.
  a. Immerse left hemisphere (or right) in 15 mL tube with 8 mL of freshly made 4% PFA for 24 h.
  b. Wash 3× with 1× cold DPBS.
  c. Immerse in 30% sucrose with 0.02% sodium azide for 2–3 days until brain sinks to bottom of tube.
  d. Embed in Tissue-Tek O.C.T. by freezing over isopentane bath immersed in liquid nitrogen and long-term store at −80°C.
5. Determine RNA integrity number (RIN) value using Step 3 10× Visium fresh frozen hemisphere.
  a. Incubate embedded hemisphere in −20°C cryostat for at least 30 min.
  b. Coronally mount fresh-frozen hemisphere to cryostat with the back of the brain positioned on the cryostat pestle (rostral to caudal).
  c. Collect three adjacent 50 μm sections (total 150 μm) into sterile 1.5 Eppendorf tube.
  d. Cover exposed sectioned tissue with Tissue-Tek O.C.T. and store in −80°C again in sealed container or plastic bag with desiccant.
  e. Isolate RNA from tissue sections using the QIAGEN kit.
  f. Once RNA is isolated, use a bioanalyzer to measure RIN proceeding with samples of a value of at least 7 (Figure 2).
6. Once RIN value is determined and tissue is ready to mount on 10× Visium slide, the next day or more, coronally re-mount fresh-frozen hemisphere from Step 5 to cryostat in the same position (rostral to caudal) as used for RIN value tissue collection (Figure 3A).
  a. Set cryostat chamber temperature to −20°C and specimen head temperature to −10°C to prevent tissue tearing (refer to problem 1 for troubleshooting).
  b. Label four 1.5 mL Eppendorf tubes to collect tissue sections for scRNAseq and determine desired range of Bregma points to collect per tube.
  c. Begin section collection at Bregma +2.245 mm for adult mice. Determine bregma locations and anatomical features with Allen Brain Atlas (atlas.brain-map.org)^12^^,^^13^^,^^14^ and P0 based on P4 interactive viewer of DevCCF.^15^
    ***Note:*** This protocol is optimized to target the striatum and cortex, thus, modify the protocol accordingly based on mouse brain regions of interest.
    ***Optional:*** Tissue samples can be scored during sectioning to reduce the amount of O.C.T per tissue and get a more accurate tissue weight for scRNA-seq. This will also help with reducing tissue curling when sectioning and help with aligning tissue into 10× Visium capture area (Figure 3B).
  d. Collect each section of tissue and count to determine distance into the brain (Figure 3C).
  e. For the first two tubes of section collection, set tissue section thickness to 50 μm, but once getting to region of interest and to reduce sectioning past desired location, set tissue section thickness for the third and fourth tubes to 10 μm.
  f. Approximately, the first tube will have 500 μm, the second and third tubes will have 300 μm and the fourth tube will have 500 μm.
  g. Use the fourth tube for proceeding with scRNAseq as it is the closest to the Visium section to match the location.
  h. Throughout the collection, mount sections to regular slides to evaluate relative position, anatomical morphology and confirm Bregma location.
7. Once arriving at a desired location for spatial transcriptomics at Bregma +0.38 mm, take an image with a brightfield microscope (i.e., EVOS) with a practice section to confirm location and improve consistency across mounted samples (Figures 3D and 3E).
8. Mount 10 μm tissue section within capture area (refer to problem 2 for troubleshooting).
  a. Mount samples randomly by genotype if have the same permeabilization time. Permeabilization time is determined with Visium Spatial Tissue Optimization (protocol CG000238) which needs to be performed before starting Spatial Transcriptomics Tissue Processing.

***Note:*** For additional guidance on mounting tissue to the Visium slide, refer to 10× Genomics troubleshooting protocols (protocol CG000240).

9. Place a finger on the backside of the capture area by gently touching the section so that it melts to the active surface of the slide.

**CRITICAL:** Do not warm up any other tissue sections on the slide.

10. Immediately place slide on the cryobar to allow section to freeze.

***Note:*** It is not recommended and is technically difficult, therefore, do not remove tissue once mounted.

11. Store slides in slide mailer sealed airtight in a plastic bag with the opening sealed with parafilm.
12. Store slide at −80°C for up to 4 weeks to avoid multiple freeze thaw cycles.
13. Store scRNAseq tissue sections at −80°C for long term storage. Avoid touching the side of the tube so that sections stay frozen to avoid freeze thaw.

***Note:*** The next steps for spatial transcriptomics and scRNA-seq can be interchangeably performed in the order desired.

### Spatial transcriptomics tissue processing with 10× Visium

**Timing: 5 h**

The next major step describes general steps used and modified based on 10× Genomics Visium protocols related to staining with immunofluorescence antibodies (protocol CG000312) and imaging (protocol CG000241). Follow the 10× guidelines for this section with minor modifications detailed below.

***Note:*** Use a sterile RNAse free workspace and tweezers when handling Visium slides to avoid impact on RNA quality.

14. Ensure that methanol (40 mL/slide) dispensed in a 50-mL standing centrifuge tube is chilled to −20°C (approximately 30 min – 1 h).
15. Place a Thermocycler Adaptor on a C1000 thermal cycler set at 37°C and equilibrate for 5 min.
16. Remove slide from −80°C and place on dry ice in a sealed container.
17. Place slide on Thermocycler Adaptor with active surface facing up and incubate 1 min at 37°C.
  a. Do not close thermal cycler lid and maintain machine at 37°C.
18. Remove slide from Adaptor and completely immerse slide in prechilled methanol.
19. Methanol fix sections for 30 min at −20°C in 50 mL conical tube.
20. Place slide in Visium Slide Cassette.
21. Remove Ribonucleoside Vanadyl Complex from −20°C and place at 65°C for 5 min until solution is reconstituted to a dark green solution with no visible particulates.
  a. Allow solution to cool to ambient temperature before use and proceed immediately to making 2× and 1× Blocking Buffer and Wash Buffer on ice.
22. Vortex 1× Blocking Buffer and add 70 μl along the side of the wells to uniformly cover the tissue sections. Tap gently to ensure even coverage.
23. Incubate for 5 min at 20°C.
24. Prepare Primary Antibody Solution on ice. Pipette mix.
25. Stain using immunohistochemistry for astrocytes (Gfap, 1:200 dilution) and neurons (NeuN, 1:200 dilution) by adding 50 μl along the side of each well.
26. Incubate for 30 min at 20°C.

***Note:*** Important that concentrations of primary antibodies be optimized since many antibodies are not compatible with methanol fixed tissue and decreased antibody incubation time at 20°C. Keep concentrations consistent across time points and samples if the question is to compare proteins across ages.

27. Follow with respective secondary antibodies with 488 donkey anti-chicken (1:200 dilution) and 555 donkey anti-mouse (1:200 dilution) and nuclear stain (DAPI) and make mixture. Pipette mix. Avoid light exposure.
28. Remove Primary Antibody Solution.
29. Add 100 μl Wash Buffer along the side of each well. Remove Wash Buffer.
30. Repeat wash steps four more times for a total of five washes.
31. Add 50 μl Secondary Antibody Solution along the side of each well.
32. Incubate for 30 min at 20°C. Avoid light exposure by putting cassette in drawer or under aluminum foil.
33. Remove Secondary Antibody Solution.
34. Add 100 μl Wash Buffer along the side of each well. Remove Wash Buffer.
35. Repeat wash steps four more times for a total of five washes.
36. Dispense 3× SSC Buffer in a beaker. Remove the slide from cassette.
37. Immerse the slide 20 times in the 3× SSC Buffer. Wipe excess liquid from back of slide.
38. Add 200 μl Mounting Medium to cover tissue sections on the slide uniformly.
39. Apply the coverslip at an angle on one end of the slide. Slowly lower the coverslip, pressing down gently with forceps.
40. Immediately proceed with imaging.
41. There are multiple microscopes that can be used for imaging. Use the BZ-X Keyence microscope with consistent settings between samples and refer to 10× Genomics Imaging Guidelines (technical note CG000241).
  a. Ensure that imaging system follows recommendations: light source with wavelength range of 380–680 nm, monochrome camera, DAPI filter cube, FITC/GFP filter cube and TRITC cube, 2.18 μm/pixel minimum capture resolution and exposure times 100 milli sec-2 sec.
  b. Using the Keyence, create a stitch and area around the capture area and fiducial frame. Ensure images are saved as a multi-channel image TIFF.

***Note:*** Recommended to image as quickly as possible, less than 1 h for imaging of the entire Visium slide (4 samples).

42. Once imaging is complete, immerse slide sideways in the beaker containing 3× SSC with coverslipped surface fully sideways.
43. Keep slide in 3× SSC Buffer until coverslip slowly separate aways from the slide.
44. Remove slide from 3× SSC Buffer and immerse 1× in 3× SSC Buffer to ensure all Mounting Medium is removed. Wipe excess liquid from back of slide.
45. Place slide in a clean Visium Slide Cassette. Add 50 μl along the side of the wells.
46. To release mRNA within the tissue section, permeabilize the sections.

***Note:*** Timing depends on the tissue time point (i.e. 6 min for P0 and 4-week tissue sections, 18 min for 12-week tissue sections, determined with Visium Spatial Tissue Optimization, protocol CG000238) (refer to problem 3 for troubleshooting).

47. Perform all steps including reverse transcription and downstream processing to create Illumina sequencing-compatible libraries according to the 10× Genomics Visium User Guide (protocol CG000239, Rev E).

***Note:*** Step 47 was performed at a core facility and timing can vary depending on if facility or reader performs these steps.

### Single-cell RNA sequencing tissue processing with 10× FLEX

**Timing: 2 days**

This protocol provides details on processing and dissociation of postnatal day 0 and adult fresh frozen mouse tissue sections for RNA profiling using 10× FLEX Gene Expression for Chromium Single Nuclei/Cell sequencing (scRNAseq). Modifications for O.C.T. embedded tissue sections were informed by this 10× Genomics article.

***Note:*** Use a sterile RNAse free workspace when handling samples to avoid impact on RNA quality.

**Figure 4:**
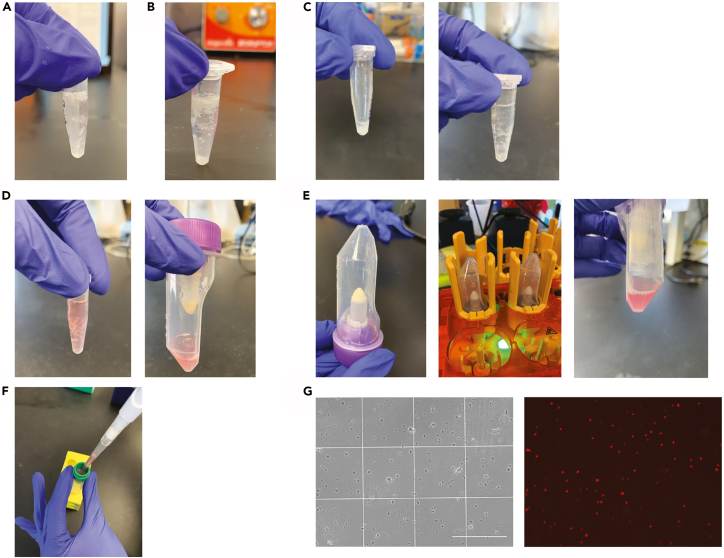
Photographic illustration of steps involved in scRNAseq for tissue section fixation and dissociation (A) Tissue sections incubated in Fixation Buffer without curls being touched by the pipette. (B) After fixation, tissue sections either float to the top of the solution or pellet in the bottom of the tube. (C) Sections will pellet during centrifugation, remove supernatant and resuspend. (D) Addition of Dissociation Solution and mix/dissociate by pipette and transfer to Miltenyi C Tube. (E) Miltenyi C Tube flipped upside down and attached to Octo Dissociator and sample dissociated after gentleMACS program. (F) For adult samples and removal of debris clusters, filter dissociated tissue through Pre-Separation Filter (30 μm) placed on a 15-ml tube keeping the P1000 at an angle when pipetting through filter. (G) Successful dissociation of tissue sections stained with AOPI stain imaged on an EVOS microscope (10× magnification, scale bar = 300 μm) and counted with a hemocytometer.

48. Depending on desired range of Bregma, use the tissue sections in tube four (sections closest to the Visium brain section) which were collected at Step 6.
49. Fix and dissociate tissue sections with the 10× Genomics Chromium Fixed RNA Profiling kit (Protocol CG000553, Rev B).
  a. Process 4–6 samples in a single round, randomly selected per round.
50. Keep tissue sections on dry ice when transferring from −80°C to the bench.
51. Weigh tissue to determine Fixation Buffer Volume.
  a. Freeze weigh boat to keep tubes frozen as well.
52. Tap tube gently on dry ice so that all frozen sections go to the bottom of the tube.
53. Fix sections with 4% PFA Fixation Buffer for 21 h at 4°C (16–24-h range depending on your protocol timing) and be consistent across rounds of fixation and dissociation.
  a. Upon adding the Fixation Buffer, some of the curls might become gelatin-like and stick to the side of the 1.5 mL tubes (Figure 4A).

**CRITICAL:** Do not attempt to detach the curls/tissue sections from the tubes. Due to the sticky nature of the sections, they may stick to pipette tips resulting in sample loss so only add the Fixation Buffer to the top of the 1.5 mL tube. Do not pipette up and down to mix, tissue sections might get stuck to pipette and result in loss of nuclei/cell yield.

**CRITICAL:** Do not agitate or mix samples during incubation.

54. After 21 h, the tissue sections may separate into sections that either float to the top of the solution or pellet in the bottom of the tube (Figure 4B).
55. Collect the tissue sections by centrifuging at 850 × *g* for 5 min at 20°C using a swinging-bucket rotor (use swinging-bucket rotor for all centrifuging steps) to increase cell recovery.
56. Remove the supernatant without disturbing the tissue pellet, leaving roughly 100 μl of supernatant to not disrupt the O.C.T. pellets (Figure 4C).
57. Add 1 mL of chilled PBS. The curls should come off the side of the tube but should not break apart and will remain intact (Figure 4C).
  a. For P0, do not pipette up and down to mix.
  b. For adult samples, pipette up and down 5× to mix and break apart the curls.
58. Collect the tissue sections by centrifuging at 850 × *g* for 5 min at 20°C using a swinging-bucket rotor to increase cell recovery.
59. Remove supernatant without disturbing the tissue pellet, leaving roughly 100 μl of supernatant to not disrupt the O.C.T. pellets.
60. Repeat the wash step one more time for a total of two washes. Add 1 mL of chilled PBS. The curls should come off the side of the tube but should not break apart and will remain intact.
  a. For P0, do not pipette up and down to mix.
  b. For adult samples, pipette up and down 5× to mix and break apart the curls.
61. Centrifuge at 850 × *g* for 5 min at 20°C.
62. Add 1 ml of chilled Tissue Resuspension Buffer.
  a. For P0 and adult samples, pipette up and down 5× to mix and break apart the curls.
63. Incubate tissue sections on ice for 10 min.
64. At the 5min mark, put RPMI + Liberase (Dissociation Solution) in 37°C bath. Warm Dissociation Solution for 10 min at 37°C before use.
  a. Dissociate using Liberase TL (0.2 mg/mL) for P0 tissue, or Liberase TH (0.2 mg/mL) for adult tissue.

***Note:*** When optimizing adult tissue and if Liberase TL does not remove sufficient debris, use Liberase TH which may be better at adult mouse brain tissue dissociation (refer to problem 4 for troubleshooting).

65. Centrifuge at 850 × *g* for 5 min at 20°C.
66. Remove Tissue Resuspension Buffer without breaking the curls leaving roughly 100 μl.
67. Add 1 mL of Dissociation Solution.
68. Pipette up and down to break up tissue as much as needed (approximately 10 times). Transfer to Miltenyi gentleMACS C tubes (Figure 4D).
69. Rinse the 1.5 mL tubes with an additional 1 ml of Dissociation Solution. Add to respective Miltenyi C tubes.
70. Place the C tube in the gentleMACS OctoDissociator with Heaters.
  a. Flip over C tube and flick bottom to ensure liquid goes to the cap (Figure 4E).
71. Run the following gentleMACS program (Figure 4E):

  gentleMACS Program

1.  temp ON

2. spin 50 rpm, 20’ 0”

3. spin-1000 rpm, 30”

4. spin-1000 rpm, 30”

5.  end

72. Centrifuge gentleMACS C tubes at 300 × *g* for 5 min at 20°C to collect dissociated cells at the bottom of the tube. Resuspend the pellet in the supernatant.
  a. Save 10 μL to check dissociation for each sample. If tissue chucks/flecks are still present in the suspension, continue to pipette mix until they are broken apart prior to filtering the suspension. The tissue chunks/flecks may clog the filter.
73. Pass the adult sample suspension through a Pre-Separation Filter (30 μm) placed on a 15-ml tube at 20°C (Figure 4F).
  a. Keep the P1000 at an angle when pipetting through filter.
  b. Rinse the 30 μm with 2 mL of PBS to recover the cells from the filter. Collect the filtrate in the same tube as the previous step.
74. Do not filter P0 samples to prevent nuclei/cell loss. Only wash C tube with 1 mL PBS, transfer to 15 mL. Repeat to bring it all up to 4 mL.

***Note:*** Filtering is not necessary since there is less debris in P0 samples compared to adult samples. Adult samples usually have more myelin which contributes to clumping and large debris.

75. Centrifuge the cell suspension at 850 × *g* at 20°C for 5 min.
76. Resuspend the adult sample pellet in 1 mL chilled Quenching Buffer, pipette mix 5 times and maintain on ice.
  a. For P0, resuspend in 500 μl for counting purposes to get a high cell count, then add another 500 μl Quenching Buffer after counting.
77. Determine cell concentration of the fixed sample using an Automated Cell Counter or hemocytometer.
  a. Count with AOPI fluorescent staining solution and dilute if necessary for target nuclei/cell load and easy counting using a hemocytometer or automated cell counter (Figure 4G) (refer to problem 4 for troubleshooting).

***Note:*** The recommended concentration for counting is approximately 1 × 10^6^ per mL (refer to problem 5 for troubleshooting). Higher or lower concentrations may increase nuclei/cell input variability. Higher concentrations will result in lower pipetting volumes while lower concentrations will result in inaccurate nuclei/cell counts.

78. Proceed immediately to 10× Genomics Single Cell Gene Expression Flex (100495 4rxns, 100496 16rxns) involving probe hybridization, library construction and sequencing with the Illumina NovaSeq 6000 system with a target sequencing depth of at least 30,000 reads/cell or store samples at −80°C for up to 6 months.
79. For long-term storage at −80°C, follow next steps.
  a. Thaw 10× Enhancer for 10 min at 65°C. Vortex and centrifuge. Keep warm and verify no precipitate before use.
  b. Add 0.1 volume of pre-warmed Enhancer to fixed sample in Quenching Buffer. For example, add 100 μl Enhancer to 1000 μl fixed sample in Quenching Buffer. Pipette mix.
  c. Make 50% Glycerol by mixing equal volume of water and 99% Glycerol, Molecular Biology Grade. Filter through 0.2 μm filter and store at 20°C.
  d. Add 50% Glycerol for a final concentration of 10%. For example, add 275 μl 50% Glycerol to 1100 μl fixed sample in Quenching Buffer and Enhancer. Pipette mix.
  e. Store at −80°C for up to 6 months.

***Note:*** Step 78 was performed at a core facility and timing can vary depending on if facility or reader performs these steps.

## Expected outcomes

The protocol developed establishes high-quality mouse brain tissue with a RIN score greater than 7 that can be used with single cell and spatial transcriptomics from the same mouse brain tissue and improves integration between datasets. Refer to 10× recommendations for quality control through the web summary since exact metrics will vary depending on the pipeline and parameters chosen and will include median genes per spot, reads mapped confidently to genome etc. For 10× Visium, the mouse brain will generate a high-quality molecular map that can also be evaluated based on Technical Note CG000290 on Sequencing Metrics & Base Composition of Visium Spatial Gene Expression Libraries. In general, sequence 50,000 minimum read pairs per spot covered with tissue, more than 50% for fraction of reads mapped confidently to genome and fraction reads in spots under tissue, and over 75% of valid barcodes. P0 tissue will cover approximately 900 Visium spots under tissue and adult samples will cover 2,500 Visium spots. For 10× FLEX, from 600 μm of tissue sections, the expected nuclei/cell yield is approximately 1.4 million cells for adult brain tissue and 928,000 cells for P0 brain tissue. These numbers are more than sufficient for the 10× Genomics recommended minimum input of 400,000 cells per hybridization for single plex and 100,000 cells for multiplex. It is recommended to generate enough material to ensure proper surveying of the regions desired and any downstream problems that may occur (i.e., loss of cells during probe hybridization).

## Quantification and statistical analysis

**Figure 5:**
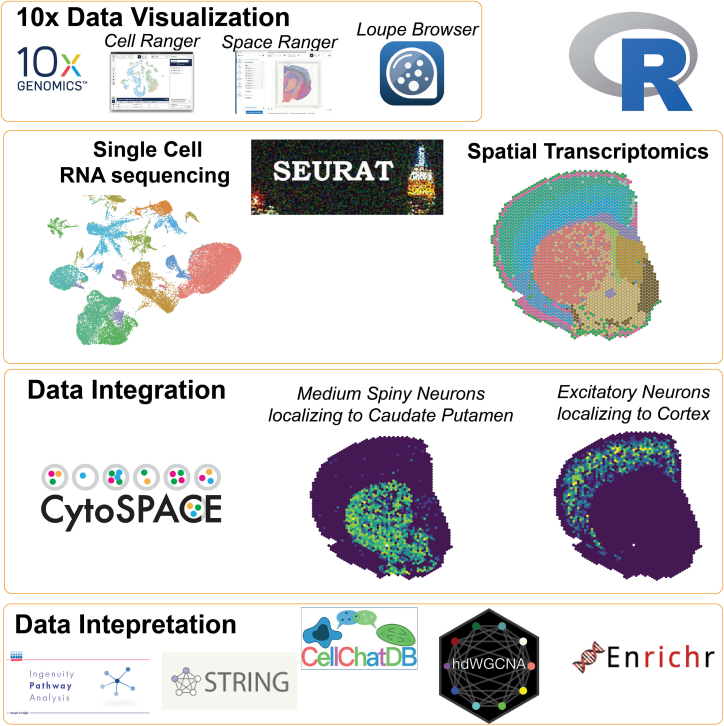
Outline of software used for data quantification, integration of scRNAseq and spatial transcriptomics and data interpretation

After generation of spatial and snRNA sequencing data, there is software (R, Seurat) for data visualization (Space Ranger, Loupe Browser, CellRanger), batch correction (Harmony^7^), data integration (CytoSPACE^8^), pathway analysis (Ingenuity Pathway analysis, Enrichr, STRING, hdWGCNA^9^), cell-cell signaling (CellChat^10^^,^^11^) and more (Figure 5). These steps will be dependent on your project study questions, tissue type and pathological states.

## Limitations

We acknowledge that Visium spatial transcriptomics has a resolution of 55 μm presenting a potential limitation to the study and findings since there are ∼1–10 cells per spot, addressed by integration with scRNAseq data. The field is constantly evolving, with technologies that include both single cell and spatial information within the same platform such as 10× Genomics Visium HD which is whole transcriptome and contains a 10 μm grid instead of a 55 μm spot size. The use of the CytAssist which is compatible with Visium HD and version 2 of Visium allows for improved resolution, standardized protocols for spatial transcriptomics and reduced technical expertise with mounting tissue to the 4 capture areas of the version 1 of Visium detailed in this protocol. There may be even more cells per spot in P0 than other timepoints based on brain size, however, we wished to evaluate this early timepoint using an unbiased whole transcriptome methodology. Further, aim to match sections precisely across samples, however, slight variations may occur due to technical constraints, which may be corrected through unbiased bioinformatic clustering. When comparing across time points, we acknowledge that P0 sections may contain more rostral regions and will contain different types of cells than 4 wk and 12 wk, furthermore, P0 is less anatomically defined and more difficult to collect identical sections. Due to technical difficulties and that it is not recommended to remove tissue once mounted; choose the best tissue for the technique despite slight variability among samples.

## Troubleshooting

### Problem 1

During cryosectioning of the mouse brain, the blade is resistant, and tissue sections show broken stripes.

### Potential solution

• Cutting temperature is too low. Ensure the O.C.T.-embedded brain is fully equilibrated to the temperature of the internal chamber of the cryostat, set at −20°C, which should take around 30 min to 1 h depending on the size of the tissue block.
• The cutting head temperature should be set at −10°C.
• 10× Genomics has extensive guidance and experience in mounting tissue to Visium slide, therefore, refer further to their Tissue Preparation Guide (protocol CG000240) for images of the whole Visium slide with tissue and how to practice mounting.

### Problem 2

For 10× Visium, the capture area is small and difficult to see, making it challenging to mount the tissue into the correct area.

**Figure 6:**
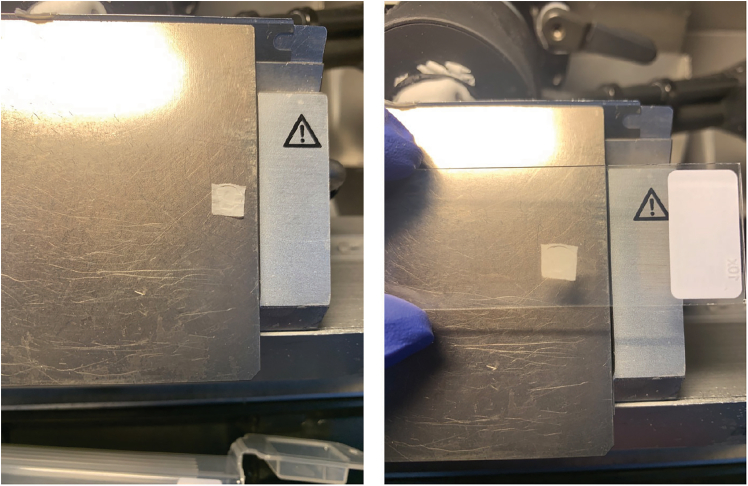
Tissue section brought to the edge of the blade holder and aligned to the Visium slide

### Potential solution

• Bring the tissue section to the edge of the base/blade holder so that you can align the edge of the capture area to the edge of the platform.
• Try to use the light reflection to better visualize the capture area (Figure 6).
• Make sure you have cleaned the base with 100% ethanol to ensure the tissue section is not attracted to the slide by static and jumps up to the slide.

### Problem 3

**Figure 7:**
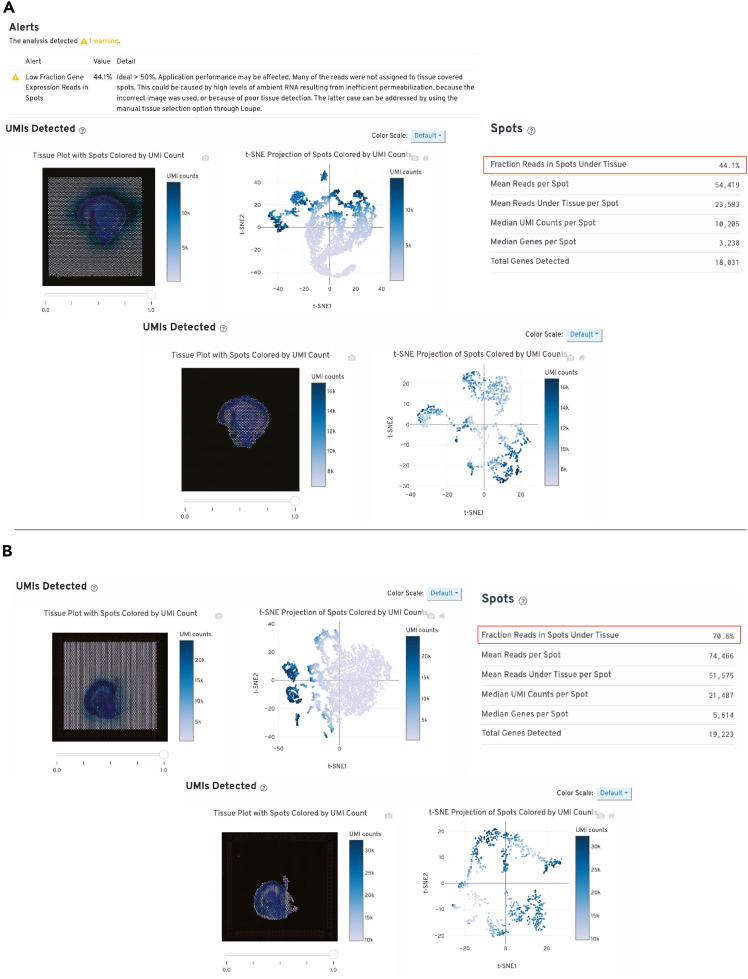
Web summary metrics generated from 10× Genomics SpaceRanger Web summaries generated from 10× Genomics Space Ranger pipeline after receiving raw data for P0 mouse tissue in Visium spatial transcriptomics step. The summary page will provide detailed information regarding data quality including “Fraction Reads in Spots Under Tissue”. To determine localization of diffused RNA and confirm that RNA is “leaking” from tissue section, rerun Space Ranger on all spots in the Visium capture area. If Fraction Reads in Spots Under Tissue is below 50%, optimization is required. (A) Unsuccessful reads in spots are most likely due to over permeabilization when releasing RNA. (B) Successful processing of P0 tissue with 10× Visium.

While in Loupe Browser, the sample for 10× Visium had less than 50% fraction of reads in spots under tissue indicating excessive diffusion of RNA (P0 as an example, Figure 7A).

### Potential solution

• Perform tissue optimization assay to determine tissue permeabilization time to release RNA on tissue determined with Visium Spatial Tissue Optimization, (protocol CG000238). It may be necessary to repeat Permeabilization Optimization to avoid over or under permeabilization.
• Ensure that all samples on the same slide have the same permeabilization time.
• 10× Genomics recommends above 50%, which will be sufficient for analysis (Figure 7B).

### Problem 4

**Figure 8:**
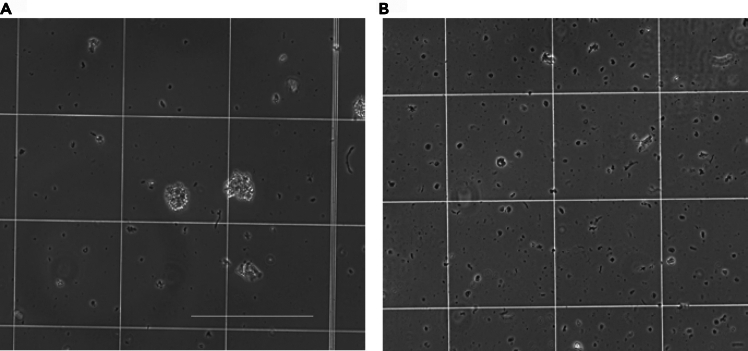
Example of unsuccessful and successful tissue section dissociation (A) Nuclei/cell clumping and sticking together when going to count on a hemocytometer. Sign that tissue has not been successfully dissociated and debris increases the chance of clogging the 10x Genomics chip for downstream processing. 10× magnification EVOS image of nuclei/cells on a hemocytometer. (B) Successful dissociation and cell dissociation. 10× magnification EVOS image of nuclei/cells on a hemocytometer. Scale bar = 300 μm.

For 10× FLEX, nuclei/cells are clumping together making it difficult to dissociate and count (Figure 8A).

### Potential solution

• After fixation and removing supernatant, make sure to pipette up and down at least 5 times for each wash to mix and break apart the curls. Adult tissue usually needs more aggressive dissociation.
• Test Liberase TH instead of Liberase TL which indicates a stronger reaction. Alternatively, test longer dissociation times and incubation in Liberase mixture or increase concentration of Liberase from 0.2 mg/mL to 1 mg/mL.
• Pass the adult sample suspension through a Pre-Separation Filter (30 μm). Cells will be individual and separate with successful dissociation (Figure 8B).

### Problem 5

There are not enough nuclei/cells to perform 10× FLEX scRNAseq.

### Potential solution

• Increase and collect tissue sections that are more rostral to the Visium section but keep consistent across samples.
• If cells are fully dissociated, do not filter since that may result in loss of nuclei/cells.

## Resource availability

### Lead contact

Further information and requests for resources and reagents should be directed to and will be fulfilled by the lead contact, Leslie M. Thompson (lmthomps@uci.edu).

### Technical contact

Technical questions on executing this protocol should be directed to and will be answered by the technical contact, Mara S. Burns (msburns@uci.edu).

### Materials availability

This study did not generate new unique reagents.

### Data and code availability

• All sequencing datasets generated as part of the original study^1^ from this protocol are publicly available in NCBI Expression Omnibus (GEO).
• The accession number for the spatial data is NCBI GEO: GSE285858.
• The accession number for the scRNA-seq data is NCBI GEO: GSE284468.
• This paper does not report original code.
• Any additional information required to reanalyze the data reported in this paper is available from the lead contact upon request.

## Acknowledgments

This protocol was supported by the following NIH grants: R35NS116872 (L.M.T.), R01NS089076 (L.M.T.), and F31NS134306-01A1 (M.S.B.). This project was supported through HD-CARE funding. M.S.B. was supported by CIRM EDUC4-12822 and ARCS Foundation Orange County. This work was possible, in part, through access to the UCI Genomics Research and Technology Hub (GRT) of the Cancer Center Support Grant (P30CA62203) and UCI Institute for Precision Health.

## Author contributions

M.S.B., R.G.L., J.C.R., and L.M.T. designed and oversaw the study. M.S.B. performed Visium up to the permeabilization step and provided the samples to the UCI GRT core for further processing, library preparation, and sequencing. M.S.B. performed scRNA-seq fixation, tissue dissociation, and sample storage and then provided the samples to the UCI GRT core for probe hybridization, library preparation, and sequencing. M.S.B. and R.M. handled the mice and perfused and isolated the brains. M.S.B. and L.M.T. wrote the manuscript. All authors read, edited, and approved the manuscript.

## Declaration of interests

The authors declare no competing interests.
